# *Culicoides paolae* and *C. circumscriptus* as potential vectors of avian haemosporidians in an arid ecosystem

**DOI:** 10.1186/s13071-018-3098-8

**Published:** 2018-10-01

**Authors:** Jesús Veiga, Josué Martínez-de la Puente, Radovan Václav, Jordi Figuerola, Francisco Valera

**Affiliations:** 1 Departamento de Ecología Funcional y Evolutiva, Estación Experimental de Zonas Áridas (EEZA-CSIC), Ctra. de Sacramento s/n, La Cañada de San Urbano, E-04120 Almería, Spain; 2 Departamento de Ecología de Humedales, Estación Biológica de Doñana (EBD-CSIC), Seville, Spain; 3 Centro de Investigación Biomédica en Red de Epidemiología y Salud Pública (CIBERESP), Madrid, Spain; 4 Institute of Zoology, Slovak Academy of Sciences, Dúbravská cesta 9, SK-84506 Bratislava, Slovakia

**Keywords:** Biting midges, *Haemoproteus*, Vector-borne, Arid area, Endophagy, Blood meal, *Coracias garrulus*

## Abstract

**Background:**

Haemosporidians are the most important vector-borne parasites due to their cosmopolitan distribution and their wide range of hosts, including humans. Identification of their vectors is critical to highlight ecologically and epidemiologically relevant features such as host specificity or transmission routes. Biting midges of the genus *Culicoides* are considered the main vectors of *Haemoproteus* spp., yet important information on aspects such as vector feeding preferences or vector-host specificity involving haemosporidian parasites is frequently missing.

**Methods:**

We assessed the abundance of *Culicoides circumscriptus* and *C. paolae* and blood sources of the latter at the nests of cavity-nesting bird species (mainly the European roller *Coracias garrulus*) and in their surroundings. We also explored the prevalence and genetic diversity of avian haemosporidians in parous females of both species.

**Results:**

Both *C. circumscriptus* and *C. paolae* were abundant in the study area and common at European roller nests. *Culicoides paolae* had a diverse ornithophilic diet, feeding on at least seven bird species. Human DNA was also detected in the blood meal of some individuals. Four *Haemoproteus* lineages, including a new one reported here for the first time, were isolated from parous females of both biting midges.

**Conclusions:**

*Culicoides circumscriptus* and *C. paolae* can play a locally important role in the transmission dynamics of *Haemoproteus* parasites in a community of cavity-nesting bird species in an arid ecosystem.

## Background

Biting midges of the genus *Culicoides* are small, cosmopolitan blood-sucking insects playing an important role as vectors of numerous viruses, filarial nematodes and protozoa affecting human, livestock and wildlife [1, 2]. *Culicoides* are regarded as the main vectors of *Haemoproteus* (class Aconoidasida, order Haemosporidia, subgenus *Parahaemoproteus*) [3]. Still, they are the least studied of the major dipteran vector groups and our knowledge of their vectorial role is biased. On one side, their participation in the transmission of livestock and human viruses has received much attention (e.g. bluetongue virus [4], African horse sickness virus [5], Oropouche virus [6]). On the other side, much less is known about their role in the transmission of avian haemosporidians. In particular, malaria-like parasites of the genus *Haemoproteus* are highly prevalent avian haemoparasites [7] with a relevant impact on the health status, longevity and fitness of their avian hosts [8–14]. *Haemoproteus* presents a high diversity in host-parasite associations [7] and it is unclear to what extent this diversity is due to host-parasite, host-vector or vector-parasite specificity [15–20]. Moreover, the vector identity and ecology of most *Haemoproteus* lineages is unknown [7, 21].

Tracing the feeding preferences (i.e. feeding patterns) of female *Culicoides* is critical to identify host-vector-parasite associations as well as ecologically and epidemiologically relevant features such as host specificity or transmission routes. Biting midges have a clear preference to feed mainly on either birds or mammals, with some species showing an opportunistic behaviour [22]. Traditionally, the feeding preferences of *Culicoides* have been assessed based on morphological characterization of the sensory structures (i.e. palps and antennae [23–25]). Other methods, such as the precipitin test [26–28], immunological assays [29, 30], and more recently, MALDI-TOF [31] and molecular tools [32, 33], have been applied to specifically identify the blood meal sources of female *Culicoides* and other insect vectors. Methods like the immunological assays are useful when the suitable hosts are suspected, which is commonly the case for the *Culicoides* surveys done in relation with livestock [34–37]. Yet, biting midges trapped in the wild may have a broad range of potential hosts, supporting the necessity to use approaches allowing the identification of a wide range of vertebrate species [22]. Studies on *Culicoides* feeding preferences in natural areas are especially scarce, although they provide a more complete view of the circulation of the blood parasites in the wild.

Here we studied the role of two common ornithophilic species of *Culicoides* in the transmission of avian haemosporidians in the driest European area, the Desert of Tabernas (south-eastern Spain). In this area, the prevalence of infection by *Haemoproteus* spp. varies between avian species, with a total absence of parasites found in adult Trumpeter finches (*Bucanetes githagineus*) while all the adult European rollers (*Coracias garrulus*) sampled showed evidence of infection [38, 39]. The haemosporidian species described for rollers in this area (*Haemoproteus coraciae*) was clustered, based on phylogenetic analysis, with other *Haemoproteus* spp. vectored by *Culicoides* [39], although the dipteran species involved in its transmission remains unidentified.

At least 81 species of the genus *Culicoides* are present in Spain [40]. In south-eastern Spain, different ornithophilic *Culicoides* species have been recorded including *Culicoides paolae*. This species, registered for first time in Spain in 2008 [41], has been frequently associated with livestock farms [34, 42, 43]. However, analysis of the sensory structures suggests an ornithophilic preference in this species [44], although the host sources of blood remain unidentified, and its role for the transmission of avian haemosporidians is completely unknown. This contrasts with other well-known, sympatric ornithophilic species such as *C. circumscriptus*, a common species in southern Spain which may be involved in the transmission of *Haemoproteus* parasites [18, 45, 46].

To assess the potential of *C. paolae* and *C. circumscriptus* as the vectors of blood parasites in a community of birds in south-eastern Spain we: (i) collected the specimens of the two species inside and in the surroundings of the nests of the European roller, one of the locally most abundant troglodytic species; (ii) identified the blood meal sources of engorged females; and (iii) studied the prevalence and genetic diversity of *Haemoproteus* parasites harboured by parous biting midge females.

## Methods

### Study area

This study was performed in an approximately 50 km^2^ area located in the Desert of Tabernas (Almería, SE Spain, 37°05'N, 2°21'W). The landscape mostly consists of open shrubland with olive and almond groves interspersed among numerous dry riverbeds (ramblas). Inhabited farms are scarce and scattered along the study area. The climate is temperate, semiarid Mediterranean with a strong water deficit during the long, hot summer months (June to September), when the absolute maximum monthly temperature is higher than 40 °C and the monthly average of the maximum daily temperatures remains above 30 °C [47]. The average annual temperature is 18 °C, with mild inter-annual oscillations of 3–4 °C and significant intra-annual fluctuations [47]. The mean annual rainfall is *c.*230 mm with high inter-annual and intra-annual variability [48].

The bird community comprises species that breed mainly in cavities in the study area (e.g. the little owl *Athene noctua*, scops owl *Otus scops*, Eurasian jackdaw *Corvus monedula*, common kestrel *Falco tinnunculus* and feral pigeon *Columba livia*), chiefly in natural holes in sandy cliffs but also in cavities in human constructions [49]. The European roller (hereafter roller) is a common breeding species in the study area where it is distributed patchily according to distinct geomorphological units [50]: (i) ramblas (dry stream channels with steep sandstone banks), which are linear, continuous geographical units separated from neighbouring ramblas by hills and human settlements; (ii) individual bridges with numerous, densely spaced cavities (*c.*2–3 m apart); and (iii) spatial aggregations of suitable nesting places, mostly trees with nest boxes but also small sandstone banks with natural cavities and isolated country houses with cavities. Wooden nest boxes have been placed in these habitat types and most rollers individuals are currently breeding in them (height × length × width: 310 × 232 × 230 mm, entrance diameter: 60 mm, with a removable upper lid to allow nest monitoring) installed on isolated eucalyptus trees, sandstone banks and isolated and deserted country houses [49, 50]. Rollers are migratory birds wintering in Africa and arriving at the breeding grounds in the study area when resident, secondary cavity-nesting birds are already settled. Eggs (mean clutch size = 4.23) [51] are incubated by both sexes [52] during *c.*21 days. Rollers rear a single brood per year [52] with fledglings leaving the nest approximately 20–22 days after hatching in the studied population [50].

### Culicoides trapping

*Culicoides* spp. specimens were trapped using two methods: sticky traps and CDC light traps. Sticky traps were placed in nest boxes occupied by rollers during the 2016 and 2017 breeding seasons (from 18 May to 4 July in 2016 and from 2 June to 18 July in 2017). Specifically, sticky traps were fixed under the upper lid of 69 nest boxes (32 in 2016 and 37 in 2017). In 2016 we took advantage of a pair of kestrels breeding in a next box close to a breeding pair of rollers, thus resembling natural nesting conditions with different cavity nesting bird species breeding in close proximity [50]. We followed the method described by Tomás et al. [53] (i.e using Petri dishes smeared with body gel-oil as a non-attractant glue) but replacing Petri dishes by white vegetal papers that were fixed by thumbtacks on the inner side of the upper lid. In 2016, these sticky traps (size = 63.6 cm^2^) were kept for three days in two periods of the breeding cycle: (i) at the end of the incubation phase (18–20 days after the first egg was laid); and (ii) during the nestling phase, when all chicks had already hatched (13–15 days after the first egg hatched). In 2017, sticky traps were only placed during the nestling stage, because most vectors were captured during this stage in 2016, and the trap size was increased (size = 175.5 cm^2^). Thus, in 2017, a first trap was set 13 days after the first egg hatched and kept for four days. Then, it was replaced by a new trap that was kept for a second period of four days. Additionally, opportunistic catches of *Culicoides* at the nests were made by hand during routine visits.

Additionally, CDC traps were set throughout the study area during 2016 and 2017. We used traps with UV light as they are recommended to attract *Culicoides* [54]. Moreover, since this study is part of a broader one aimed at studying the community of dipteran vectors, we also used incandescent light traps. Both trap types were put together and were also baited with CO_2_ in order to use as many different stimuli as possible. Dry ice was used as source of CO_2_ (1 kg of dry ice per night and pair of traps to ensure the continued emission of CO_2_ until the collection of the traps at dawn). Thus, 20 pairs of CDC traps (each pair formed by one trap with incandescent light and one with UV light, *c.*50 cm apart from each other, both baited with CO_2_) were set all over the study area and in the main breeding habitats of the roller, namely trees, ramblas and bridges (see above), so that eight traps were located on ramblas, eight on trees and four on bridges during 2016 and 2017. The traps were powered by a 6 V battery of 12 Ah. The trapping sessions were adjusted according to the breeding season of rollers and the moon calendar, so that traps were active on the days during or close to the period of the new moon (reducing the effect of ambient light [55]), and avoiding windy nights. In 2016 we placed one group of 10 pairs of traps from 8 June to 10 June and a second group of 10 trap pairs from 7 July to 8 July. In 2017, all 20 trap pairs were set from 22 June to 1 July. Most traps (82.5%) were set before dusk or shortly after and were removed after sunrise. Captured insects were moved to the Estación Experimental de Zonas Áridas and frozen in 70% ethanol until identification.

### Morphological identification

Biting midges were identified to the species level based on González & Goldarazena [56] and Mathieu et al. [57] taxonomic keys under a Zeiss Discovery V8 stereomicroscope. *Culicoides circumscriptus* and *C. paolae* were the most abundant biting midges at the nests (see Results), and individuals of these species collected at the nests and with CDC light traps were analysed for blood meal origin (engorged females) or *Haemoproteus* detection (parous females). Engorged females were identified based on the presence of blood remains in the abdomen. The abdomen of each *C. paolae* engorged female was separated from the head-thorax using sterile tips on chilly Petri dishes and, subsequently, maintained in individual vials. Diet analyses were restricted to *C. paolae* as only two engorged *C. circumscriptus* females were captured. Parous females were identified based on the presence of burgundy-red pigmented abdomen that develops during the first gonotrophic cycle [58]. As previous studies have reported a low prevalence of avian haemosporidians in *Culicoides* from southern Spain ([46], our unpublished observations], parous females were grouped in pools from 1 to 11 individuals according to species, date and site of capture.

### DNA extraction and molecular analyses

Genomic DNA from the abdomen of each engorged *C. paolae* females and biting midge pools was extracted using the DNeasy Blood and Tissue® kit (Qiagen, Hilden, Germany) following company specifications. Negative controls (reagents without a template) were used to detect possible contaminations. DNA was stored at -20 °C until PCR amplification. To confirm the morphological identification of *Culicoides* species, we amplified a 658 base pair (bp) fragment of the mitochondrial cytochrome *c* oxidase 1 (*cox*1 gene, barcoding region) of four individuals following Gutiérrez-López et al. [59]. The vertebrate hosts of *Culicoides* females were identified by amplification of a fragment of 758 bp of the vertebrate *cox*1 gene following Alcaide et al. [32]. Finally, the presence and identity of *Haemoproteus* and *Plasmodium* spp. were assessed for the pools of parous female *Culicoides* specimens using the protocol by Hellgren et al. [60]. Parasite determination was conducted at least twice per sample to avoid false negative results [61]. The presence of amplicons was verified on 1.8% agarose gels. Positive amplifications were sequenced using the Macrogen laboratories sequencing service (Madrid, Spain) and sequences were edited using the software Sequencher™ v.4.9 (Gene Codes Corp, Ann Arbor, MI, USA).

The identity of *Culicoides* species and their vertebrate hosts were established by comparison with sequences deposited in GenBank DNA sequence database (National Center for Biotechnology Information BLAST) or the Barcode of Life Data Systems (BOLD). The molecular identification of two female *C. paolae* and two female *C. circumscriptus* confirmed the morphological identifications. Vertebrate species were confirmed if agreement was ≥ 98% with deposited sequences. Parasite lineages and morphospecies were identified by BLAST comparison with the sequences available in GenBank and MalAvi [62].

### Statistical analyses

The abundance of parous *C. paolae* and *C. circumscriptus* captured in CDC traps were analysed with a generalized linear mixed model (GLMM) with the negative binomial distribution of errors. Year (2016 and 2017) and biting midge species were included as independent variables. Scaled and centred date of sampling was included in a GLMM as a covariate. The number (log-transformed, scaled and centred) of blood-feeding parasitic dipterans captured per pair of traps was included as an offset variable to correct for their abundance in each sampling point. Trap location, identical during both years, was included as a random factor. The interaction between sampling date and *Culicoides* species was introduced to explore a seasonal effect in the capture of the two species. One outlier due to the capture of 94 parous *C. paoale* was detected and the analyses were run with and without this datum. Given that the results obtained were qualitatively comparable, we report the analysis including this datapoint.

The prevalence of *Haemoproteus* spp. in *Culicoides* pools was estimated considering variable pool sizes and 100% test specificity and sensitivity following Sergeant [63]. Statistical analyses were performed using the R environment [64] with the *lme4* and *effects* packages [65, 66].

## Results

### Abundance of *Culicoides* spp. in avian nests

Overall, 57 *Culicoides* spp. were collected in avian nest during both years (*n* = 42 in 2016 and *n* = 15 in 2017, Table 1). From the 57 captures, four individuals were collected opportunistically in the nest and 53 were collected by sticky traps. In addition, Simuliidae (*n* = 230) and Phlebotominae (*n* = 105) were other blood-feeding dipterans collected with the sticky traps at the nests.

**Table 1:** Abundance, mean ± SD, and range (in parentheses) for *Culicoides* spp. (overall data set) and for the subset of parous and engorged females of *C. paolae* and *C. circumscriptus* trapped in avian nests and their surroundings during 2016 and 2017

	Inside nest		Outside nest	
	2016 (*n* = 33^a^ nests)	2017 (*n* = 37 nests)	2016 (*n* = 20 trap pairs)	2017 (*n* = 20 trap pairs)
*Culicoides* spp*.*	42	15	3585	4179
	1.27 ± 4.38	0.41 ± 0.90	179.25 ± 160.77	208.95 ± 187.31
	(0–25)	(0–4)	(0–423)	(2–380)
*C. paolae* parous	26	1	77	180
	0.79 ± 3.56	0.03 ± 0.164	3.85 ± 6.79	9 ± 20.59
	(0–20)	(0–1)	(0–27)	(0–94)
*C. circumscriptus* parous	3	8	34	50
	0.09 ± 0.29	0.22 ± 0.75	1.70 ± 3.42	2.5 ± 3.01
	(0–1)	(0–4)	(0–15)	(0–11)
*C. paolae* engorged	6	0	14	16
	0.18 ± 0.58		0.7 ± 0.92	0.8 ± 1.06
	(0–2)		(0–3)	(0–3)
*C. circumscriptus* engorged	0	0	0	2
				0.1 ± 0.45
				(0–2)

^a^All nests corresponded to European roller nests with the exception of a single common kestrel nest sampled in 2016

The most abundant biting midges were *C. paolae* (57.9%, 33 out of 57) and *C. circumscriptus* (22.8%, 13 out of 57). Twenty-seven *C. paolae* specimens were parous and six were engorged, whereas 11 *C. circumscriptus* specimens were parous, two nulliparous (not included in Table 1) and no engorged individual was captured. Twenty-two out of the 32 *C. paolae* collected in 2016 were captured in a common kestrel nest.

### Abundance of *Culicoides* spp. in CDC traps

Overall, 7764 *Culicoides* spp. were captured using CDC traps (Table 1). Of them, 341 were parous females of *C. paolae* and *C. circumscriptus*, representing 4.4% of the total *Culicoides* spp. specimens captured (Table 1). The average number of parous females per pair of traps was 3.8 of *C. paolae* and 1.7 of *C. circumscriptus* in 2016 and 9 of *C. paolae* and 2.5 of *C. circumscriptus* in 2017. Furthermore, 30 engorged females of *C. paolae* and two engorged females of *C. circumscriptus* were also captured (Table 1).

**Fig. 1:**
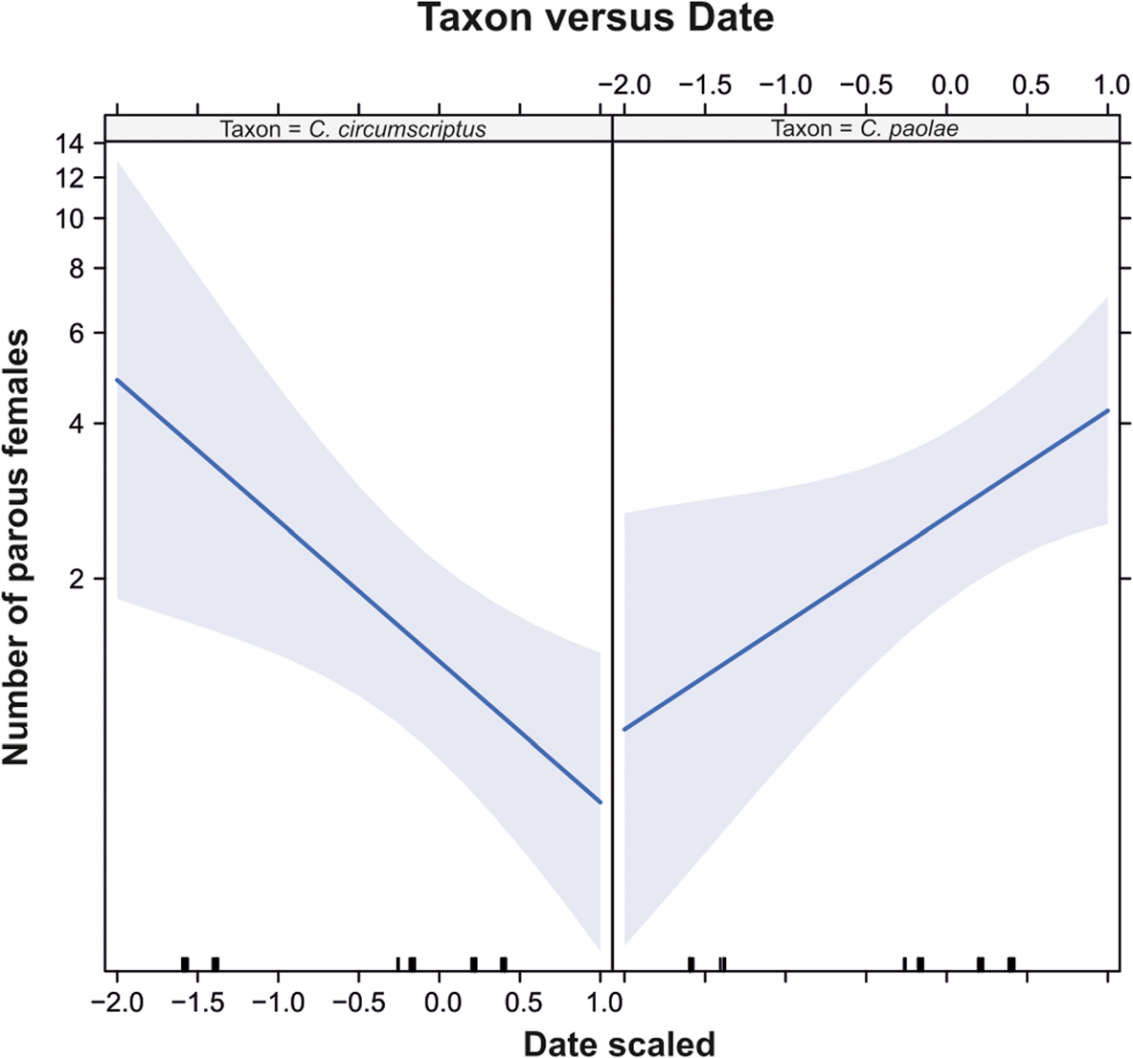
Relationship between capture date and abundance of parous females of *Culicoides circumscriptus* (estimate ± SE = -0.63 ± 0.23, *P* < 0.01) and *C. paolae* (estimate ± SE = 0.48 ± 0.21, *P* = 0.022) captured with CDC traps in south-eastern Spain during 2016 and 2017. Date of capture was scaled and centred. Lines represent fitted values with shaded regions showing areas delimited by 95% confidence intervals

The abundance of parous biting midges (*C. paolae* and *C. circumscriptus*) was greater in 2017 and decreased through the breeding season. *Culicoides paolae* was significantly more abundant than *C. circumscriptus* (Table 2). The interaction between *Culicoides* species and sampling date was also significant (Table 2), because parous *C. paolae* was more abundant late in the roller breeding season and parous *C. circumscriptus* was more abundant early in the season (Fig. 1).

**Table 2:** Results of a generalised mixed model analysing the abundance of parous *Culicoides paolae* and *C. circumscriptus* collected using CDC traps in relation to year (2016, 2017), date of capture, and the interaction between date of capture and the species of *Culicoides* biting midges

Fixed effects	Estimate	SE	*z*-value	*P*
Intercept	0.043	0.28	0.16	0.88
Species (*C. paolae*)	0.64	0.29	2.26	0.024
Date	-0.63	0.23	-2.71	0.007
Year (2017)	0.56	0.29	1.92	0.053
Species (*C. paolae*)*Date	1.10	0.31	3.57	0.0004

### *Culicoides paolae* feeding patterns

Blood meals of 21 (58.3%) out of the 36 engorged *C. paolae* were successfully identified. Of them, the six females captured in avian nests fed on the species breeding in these nest boxes (two on common kestrels and four on rollers) (Table 3). *Culicoides paolae* females captured with CDC traps (*n* = 15) fed mainly on birds (66.7% of the identified blood meals) corresponding to five different species including cavity-nesting and open-nesting species. Finally, human DNA was found in five (33.3%) out of 15 *C. paolae* collected with CDC traps (Table 3).

**Table 3:** Hosts of *C. paolae* based on the molecular identification of blood meal origin. The number of successfully identified blood meals is shown for each species

	Host	Cavity/open nester	No. of successful amplifications
Inside nests	European roller (*Coracias garrulus*)^a^	Cavity nester	4
	Common kestrel (*Falco tinnunculus*)^a^	Cavity nester	2
CDC traps	Humans (*Homo sapiens*)	–	5
	House sparrow (*Passer domesticus*)	Cavity nester	4
	Common blackbird (*Turdus merula*)	Open nester	3
	Eurasian hoopoe (*Upupa epops*)	Cavity nester	1
	Eurasian collared dove (*Streptopelia decaocto*)	Open nester	1
	Common linnet (*Linaria cannabina*)	Open nester	1

^a^The blood in the abdomen of the biting midge belonged to the avian species breeding at the nest where the biting midge was collected

### Prevalence and identification of haemosporidian parasites

The prevalence of *Haemoproteus* spp. was 4.4% (95% CI: 1.39–9.95%, *n* = 95 individuals) and 0.7% (95% CI: 0.12–2.21%, *n* = 284 individuals) for *C. circumscriptus* and *C. paolae* pools, respectively. Overall, four *Haemoproteus* and one *Plasmodium* lineages were found. Of them, the three *Hemoproteus* lineages, TURDUS2, GAGLA03 (= GAGLA05, both sequences with equal coverage and identity) and AEFUN03, and the *Plasmodium* lineage SYAT05 (*Plasmodium vaughani*), were identified with 100% coverage and identity. The three *Haemoproteus* lineages, TURDUS2, GAGLA03 (=GAGLA05) and AEFUN03, were isolated from *C. circumscriptus* specimens (Table 4). The *Haemoproteus* lineage TURDUS2 and the *Plasmodium* lineage SYAT05 were isolated from *C. paolae* specimens (Table 4). In addition, a new lineage (CUPAO-01, GenBank: MH237967) was isolated from a *C. paolae* specimen. This lineage showed 93% overlap and 99% similarity with the *Haemoproteus coraciae* lineage H1CG.1 (GenBank: KU297278) (Table 4). In fact, six nucleotide bases differed between both lineages.

**Table 4:** Molecular identification of haemosporidians in pools of parous *C. circumscriptus* and *C. paolae* females trapped in avian nests and surroundings. Lineages and accession numbers from GenBank sequences showing the highest percentage of coverage and identity to those found in this study are shown. Previous information regarding these sequences is reported including the parasite morphospecies (when described), avian hosts and potential insect vectors (in bold) according to information of the reported sequences

Pool code	Host in this study	Closest lineages (morphospecies)	GenBank ID	Potential avian hosts and vectors described	Coverage/identity (%)
N7c35 (Accession no.: MH237967)	*C. paolae*	H1CG.1 (*Haemoproteus coraciae*)	KU297278	*C. garrulus*	93/99
N26c1 NFc1	*C. circumscriptus* *C. paolae*	Turdus2 (*H. minutus*)	MF625183 KM361485 KJ488583 KC818452 JN819398 JN819388 JN819383 HQ398208 DQ630013 DQ060772	*E. rubecula* *G. glandarius* *M. striata* *T. merula* *T. assimilis* *T. icterocephala* *B. lineola*	100/100
NEc3 NEc4	*C. circumscriptus*	GAGLA05 GAGLA03 (*Haemoproteus* sp.)	KX831071 KJ488735 GU085197 MF594402 MF095639	*G. glandarius* ***C. circumscriptus***	100/100
AGALM4	*C. circumscriptus*	AEFUN03 (*Haemoproteus* sp.)	KP715101	*A. funereus*	100/100
N31c2	*C. paolae*	SYAT05 (*Plasmodium vaughani*)	MF817773 MF347700 KJ488789 JF411406 AB477124 DQ847271	*C. caeruleus* *S. maurus* *S. unicolor* *S. atricapilla* *T. merula* *T. migratorius* *T. philomelos* *T. viscivorus* ***C. pipiens***	100/100

## Discussion

This study reveals that *C. circumscriptus* and *C. paolae* are common endophagous insects at the nests of cavity-nesting species, with *C. paolae* being identified for the first time, using identification of blood meals, as a potential vector of avian haemosporidians. This assertion is supported by detecting a high diversity of avian hosts including cavity-nesting and open-nesting species and the identification of avian haemosporidian parasites for *C. paolae*.

Whereas *C. circumscriptus* is common in Spain [56, 67–72], *C. paolae* was detected for the first time in 2008 [41]. It has been proposed that the latter species was introduced into Europe by Columbus’s travels from America five centuries ago [44, 73]. In spite of some morphological differences, *C. paolae* is very similar to the American *Culicoides jamaicensis* [44] and a recent phylogenetic study related the former species with *Culicoides* from the New World [73]. Nowadays, in addition to Spain, where *C. paolae* is currently expanding its distribution range [41], this biting midge is the most widespread and abundant species of all *Culicoides* in Malta [34] and one of the most abundant species in central Tunisia [74] and Sardinia [75], where its importance on the local transmission of avian vector-borne pathogens should be considered. *Culicoides paolae* is commonly found near livestock farms [34, 74, 75], but according to our results, this species may also be widespread in the wild and, at least for the study period, it is even more abundant than *C. circumscriptus* (Tables 1, 2). Data from two breeding seasons suggest that the two species exhibit different phenologies, *C. paolae* being more abundant late in the roller breeding season whereas the opposite is true for *C. circumscriptus*. Furthermore, whereas the ability of some ornithophilic biting midges to feed inside enclosed places (endophagy) has been previously shown [39, 53, 69, 76], to our knowledge this is the first time that endophagy has been recorded for *C. paolae*.

*Culicoides paolae* is defined as ornithophilic according to its sensory structures [44]. Here we provide for the first time, unequivocal identification of its hosts, including seven different bird species within the study area. This broad spectrum of hosts has already been described for other ornithophilic *Culicoides* species [22, 77]. Interestingly, some of the host species are not particularly abundant in the study area, suggesting a remarkable feeding range of this biting midge and excellent host-searching abilities. Nonetheless, a greater effort in sampling engorged females *C. paolae* together with an analysis of the bird community composition around the traps is still necessary for a better knowledge of host selection by this dipteran. Our results also suggest that *C. paolae* could feed on humans. Even though we tried to minimize the risk of contamination, we did not type the human-positive samples with DNA samples of the experimenters (e.g. [78]). Thus, we cannot discard the possibility of sample contamination. Nonetheless, other ornithophilic species like *C. kibunensis* [79, 80], *C. circumscriptus* [81], or *C. pictipennis* [82], have previously been reported to feed on humans. The broad range of hosts could help biting midges to face environmental changes [79], and in our case, it could have facilitated the establishment of *C. paolae* in a new area.

DNA from four *Haemoproteus* lineages and one *Plasmodium* lineage was detected in parous *C. paolae* and *C. circumscriptus*. Even though multiple *Plasmodium* lineages have been molecularly detected in *Culicoides* [18, 46], this does not imply vector competence [83]. *Plasmodium* is mainly transmitted by *Culex* mosquitoes [7] and our result could simply reflect the presence of abortive stages of *P. vaughani* in *C. paolae* [84].

We isolated four different *Haemoproteus* lineages from six pools of *Culicoides* females. A lineage of *H. minutus* (TURDUS2) was detected both in *C. paolae* and *C. circumscriptus*. This is a geographically widespread lineage (northwest Africa, northwest Iberia, Transcaucasia and western Greater Caucasus) infecting different avian species, with *Turdus merula* probably playing a central role as reservoir [85]. Additionally, GAGLA03 (=GAGLA05) was previously isolated in Bulgaria from *Garrulus glandarius* [86], *C. circumscriptus* in Spain ([46], this study) and Turkey (GenBank: MF594402 and MF095639). The lineage AEFUN03 that had been only detected previously in *Aegolious funereus* [87] was found in *C. circumscriptus* in south-eastern Spain. This bird species is absent from the study area and probably this *Haemoproteus* lineage is infecting another locally abundant owl (e.g. little owl *Athene noctua*). Finally, we also detected a new *Haemoproteus* lineage highly similar (99% similarity) to the one corresponding to the haplotype H1CG.1 (identified as *H. coraciae*), which was detected previously in the same roller breeding population by Václav et al. [39]. Microscopic examination of smears suggested that this lineage might correspond to the species *Haemoproteus coraciae* [39], a parasite identified in rollers in Bulgaria [88] and Kazakhstan [89]. For the case of avian malaria parasites and related haemosporidians, different lineages are described with differences of a single nucleotide base in their sequences [62]. However, different lineages showing few differences may correspond to the same parasite morphospecies. Thus, it is likely that the new lineage reported here (H1CG.1) corresponds to the *H. coraciae* morphospecies*.* Further analyses are necessary to confirm this possibility. *Haemoproteus coraciae* were widely prevalent in adult rollers and also present in nestlings, suggesting the presence of a competent vector in the breeding area [39]. Václav et al*.* [39] pointed out that the detection of a *Haemoproteus* species only infecting adult rollers was intriguing because all the *Culicoides* species studied by Bobeva et al. [82] were feeding on a wide range of avian host. Our results suggest that *C. paolae* may be a competent vector for *H. coraciae* probably playing a role on the transmission of locally circulating parasites that could be amplified by the migratory behaviour of rollers. Further analyses are necessary to confirm the vector competence of this *Culicoides* species for the transmission of the lineages isolated here [83].

The prevalence of *Haemoproteus* in *C. circumscriptus* in the study area (4.4%) is slightly lower than the one observed in central Spain (16.7% [18]) and southwestern Spain (10.3% [46]), yet it is higher than the prevalence found in the sympatric *C. paolae* (0.7 *vs* 4.4%). On the other hand, *C. paolae* is seemingly locally more abundant than *C. circumscriptus* both at the nests and in their surroundings. Therefore, both species could play an important role in the transmission dynamics of haemosporidian parasites in the study area. Nevertheless, other factors such as the efficiency of parasite transmission or seasonality in vector abundance should be considered. Concerning the latter, our study reveals that differential exposure of the hosts to individual biting midge species along the season is worth studying to fully understand the risk of haemosporidian transmission by each species.

## Conclusions

Vectors for most haemosporidians are unidentified [7, 90] and thus parasite-vector associations remain an enigmatic aspect of haemosporidian parasite ecology [17, 19, 91]. Here, we provide valuable information about the *Haemoproteus* lineages potentially transmitted by two biting midges species. *Culicoides paolae* and *C. circumscriptus* were abundant both at the nests of cavity bird species and in their surroundings, with seasonal differences in abundance during the study period. We assessed the ornithophilic diet of *C. paolae* that fed on at least seven bird species and possibly also on humans. Both biting midge species harboured several *Haemoproteus* lineages. These findings provide an important first step towards the identification of *C. paolae* and *C. circumscriptus* as potential vectors of avian haemosporidian parasites.

## Abbreviations

MALDI-TOF: matrix-assisted laser desorption/ionization time-of-flight mass spectrometry
CDC: Centers for Disease Control
GLMM: Generalized linear mixed effects models
